# Involvement of Cathepsins in Innate and Adaptive Immune Responses in Periodontitis

**DOI:** 10.1155/2020/4517587

**Published:** 2020-03-31

**Authors:** Xu Yan, Zhou Wu, Biyao Wang, Tianhao Yu, Yue Hu, Sijian Wang, Chunfu Deng, Baohong Zhao, Hiroshi Nakanishi, Xinwen Zhang

**Affiliations:** ^1^The VIP Department, School and Hospital of Stomatology, China Medical University, Liaoning Provincial Key Laboratory of Oral Diseases, Shenyang 110002, China; ^2^Department of Aging Science and Pharmacology, Faculty of Dental Science, Kyushu University, Fukuoka 812-8582, Japan; ^3^OBT Research Center, Faculty of Dental Science, Kyushu University, Fukuoka 812-8582, Japan; ^4^Center of Implant Dentistry, School and Hospital of Stomatology, China Medical University, Liaoning Provincial Key Laboratory of Oral Diseases, Shenyang 110002, China; ^5^Department of Pharmacology, Faculty of Pharmacy, Yasuda Women's University, Hiroshima 731-0153, Japan

## Abstract

Periodontitis is an infectious disease whereby the chronic inflammatory process of the periodontium stimulated by bacterial products induces specific host cell responses. The activation of the host cell immune system upregulates the production of inflammatory mediators, comprising cytokines and proteolytic enzymes, which contribute to inflammation and bone destruction. It has been well known that periodontitis is related to systemic inflammation which links to numerous systemic diseases, including diabetes and arteriosclerosis. Furthermore, periodontitis has been reported in association with neurodegenerative diseases such as Alzheimer's disease (AD) in the brain. Regarding immune responses and inflammation, cathepsin B (CatB) plays pivotal role for the induction of IL-1*β*, cathepsin K- (CatK-) dependent active toll-like receptor 9 (TLR9) signaling, and cathepsin S (CatS) which involves in regulating both TLR signaling and maturation of the MHC class II complex. Notably, both the production and proteolytic activities of cathepsins are upregulated in chronic inflammatory diseases, including periodontitis. In the present review, we focus on the roles of cathepsins in the innate and adaptive immune responses within periodontitis. We believe that understanding the roles of cathepsins in the immune responses in periodontitis would help to elucidate the therapeutic strategies of periodontitis, thus benefit for reduction of systemic diseases as well as neurodegenerative diseases in the global aging society.

## 1. Introduction

Immune responses, including the innate and adaptive immune responses, are components of a defensive mechanism which has become increasingly specialized with evolution. The human immune system is able to generate great quantities of specialized cells and molecules capable of recognizing and eliminating an apparently unlimited diversity of foreign invaders by functioning cooperatively [1]. Of note, although these specialized cells and molecules accumulate at inflammatory sites to efficiently remove invading agents, they may also amplify the inflammatory response and impair the surrounding tissues [2]. Therefore, under the inflammatory conditions, the inflammatory-responsive cell-related immune responses must be tightly controlled. Periodontitis is a chronic infectious disease whereby chronic inflammation of the periodontium involves interactions among bacterial products, numerous cell populations, and inflammatory mediators. The initiation of periodontitis might be attributed to dental plaques and complex and diverse microbial biofilms that form on the teeth. In particular, substances released from these biofilms, including lipopolysaccharides (LPS), antigens, and other virulence factors, gain access to the gingival tissues. As a result, the innate and adaptive immune responses are elicited, thus leading to the activation of host defense cells. Collectively, inflammatory mediators, which comprise cytokines and proteolytic enzymes, induce tissue destruction and bone resorption [3].

Cathepsin, a term derived from the Greek word kathepsein (meaning to digest), is a protease that is functionally active in a slightly acidic environment. There are 11 human cysteine cathepsin isoforms, referred to as B, C, F, H, K, L, O, S, V, X, and W [4]. Cathepsins are primarily intracellular enzymes responsible for nonspecific bulk proteolysis in the endosomal/lysosomal system, which degrades both intracellular and extracellular proteins [5]. However, cathepsins are involved in producing immune modulators by the limited proteolysis processing. For example, cathepsin B (CatB) involves in the production of interleukin-1*β* (IL-1*β*) [6, 7] and TNF-*α* [8] and cathepsin K (CatK) involves in toll-like receptor 9 (TLR9) activation [9]. In addition, cathepsin S (CatS) mediates TLR signaling transduction and regulates major histocompatibility complex (MHC) class II-dependent CD4^+^ T-cell activation [10]. Indeed, both the production and proteolytic activities of CatS are upregulated under conditions closely related to chronic inflammatory diseases, including periodontitis [11]. In the present review, we summarize the current knowledge on the involvement of cathepsins in regulating innate and adaptive immune responses in periodontitis. Given the roles of cathepsins in immune/inflammatory responses, the regulation of cathepsins will be helpful for the management of cellular immune responses in patients with periodontitis, and thus beneficial to prevent and relax the systemic diseases as well as neurodegenerative diseases in the global aging society.

## 2. Innate and Adaptive Immune Responses in Periodontitis

### 2.1. Periodontal Pathogens and TLRs

Periodontitis is caused by specific bacterial infection. The bacterial species living in polymicrobial biofilms or below the gingival margin proliferate largely as a result of the inflammation initiated by specific subgingival species. It is widely accepted that specific microorganisms are associated with specific periodontal diseases, and the search for the periodontal pathogens responsible for distinct periodontal conditions is under way [12, 13]. The “red complex,” which is composed of the pathogens *Porphyromonas gingivalis*, *Tannerella forsythia*, and *Treponema denticola*, has been shown to exist in the biofilms where progressive periodontitis is shown [14]. Bacterial components, such as LPS, peptidoglycans, lipoteichoic acids, proteases, and toxins, which stimulate the inflammatory activity, can be detected in the biofilms on the surface of the tooth [15]. Antigens and toxic products released by bacteria, such as LPS and peptidoglycans, are identified by TLRs on the surface of host cells and can trigger cellular signaling of host cells [16]. On the host side, the diverse inflammatory cell types and resident cells of the tissues respond to bacterial infection and induce cellular immune responses that are innate and adaptive immune responses [17]. TLRs can detect multiple pathogen-related molecular patterns, including LPS, bacterial lipoproteins, lipoteichoic acids, flagellin, CpGDNA of bacteria and viruses, double-stranded RNA, and single-stranded viral RNA [18]. To date, 11 different TLRs (TLR1–11) have been identified [16]. It has been known that lipopolysaccharides from *Porphyromonas gingivalis* (*P. gingivalis* LPS) is recognized by TLR2/TLR4 [19, 20] while flagellin in *Tannerella forsythia* is recognized by TLR5 [21]. The TLR pathway is crucial in the immune responses in periodontitis because TLRs are bound to their corresponding antigens, which triggers intracellular immune responses to produce inflammatory-related mediators [22].

### 2.2. Macrophages in Periodontitis

Macrophages are myeloid cells of hematopoietic origin, which play important role in the local immune responses [23]. The major functions of macrophages include elimination of invading bacteria, recruitment of immune cells to the site of infection, production of cytokines and chemokines, and activation of the adaptive immune response through TLR signaling [22]. The functions of activated macrophages are regulated by mediators produced by T cells. The classically activated macrophages (M1) are regulated by interferon (IFN)-*γ* and LPS [24], while alternatively activated macrophages (M2) are produced in response to IL-4 or IL-13 [25]. M2 macrophages have been shown to play a role in relief of inflammation and thus have decreased capacity to produce cytokines. Macrophages express high levels of TLR2 and TLR4 [26], which shift to the M1 phenotype in gingival tissues of patients with chronic periodontitis [27]. Interestingly, M1 macrophages can transform into M2 macrophages by exosomes derived from gingival mesenchymal stem cells, which is beneficial for suppressing the immune responses [28].

Accumulating studies have demonstrated that macrophage serves as an important route for local immune responses in periodontitis because macrophage can produce multiple proinflammatory mediators, including tumor necrosis factor (TNF-*α*) and IL-1*β* and IL-6 through activating the nuclear factor kappa-light-chain-enhancer of activated B (NF-*κ*B) pathway [29]. In addition to the contribution to innate immune response, TNF-*α* also serves as a significant route for alveolar bone resorption in periodontitis for inducing the differentiation and activation of osteoclasts [30]. IL-1*β* activates generation of matrix metalloproteinase (MMP)-9 in periodontitis [31] and promotes the expression of the receptor activator of nuclear factor kappa-B ligand (RANKL), contributing to osteoclastogenesis in periodontitis [32, 33]. A recent report has shown that IL-1*β*-expressed inflammatory macrophages produce amyloid *β* (the main component of the hall marker in the brain of AD patients) in the gingival tissues of the patients with periodontitis as well as in the liver of mice after chronic systemic *P. gingivalis* infection, indicating that inflammatory macrophages in periodontitis may contribute to neurodegenerative diseases such as AD [34]. IL-6 triggers the generation of vascular endothelial growth factor (VEGF) and MMP-1, making great contribution to the development of periodontitis [35] and synergic effects of IL-1*β* and IL-6 upregulate MMPs, contributing to the tissue destruction in periodontitis by collagen degradation and bone resorption [36]. It is noted that chemical ablation of macrophages in mice prevents the *P. gingivalis*-induced alveolar bone resorption, demonstrating important roles for macrophages during periodontitis [37].

### 2.3. Dendritic Cells in Periodontitis

Dendritic cells (DCs) are located adjacent to the epithelia of blood vessels and the mucosa that shield soft tissues from microbial invasion. Bacteria or antigen-antibody complexes of bacteria or bacterial products that breach the epithelia and mucosa will encounter DCs and induce DC responses. DCs are typically the first immune cells to encounter and respond to invading microorganisms [38]. The nature of this DC response is essential in determining the type of acquired immune response that is induced. Adaptive immune response relies on the formation of appropriate peptides from foreign proteins and the consequent presentation on MHC I or II complexes [39], and DCs are able to initiate the elimination of the MHC II-associated chaperone invariant chain (Ii) from MHC II [40, 41]. Upon the stimulation by pathogens, such as *P. gingivalis*, antigen presentation by DCs leads to the activation and subsequent differentiation of CD4^+^ T cells [36]. The unique immune-stimulatory activity of DC stems from their ability to efficiently capture and present antigens and optimally express cytokines after *P. gingivalis* LPS stimulation [42]. Naïve DCs are found in large numbers in gingival tissues, and DCs are matured in the inflamed gingival tissues from patients with periodontitis [43] which particularly serve as immune stimulators [44]. Considering the roles of DCs for cytokine production and differentiation of CD4^+^ T cells, DCs are reckoned to be the bridge between the innate and adaptive immune responses in periodontitis.

### 2.4. T Cells in Cellular Immune Responses

T cells can be activated in response to oral bacterial antigens [45], and CD4^+^ T cells play key roles in the development of a mouse model of periodontitis [46]. CD4^+^ T-cell-dependent responses are initiated by the recognition of MHC class II peptide complexes on antigen-presenting cells (APCs), such as DCs and macrophages. During the process, the invariant chain is sequentially degraded to leupeptin-induced peptide 10 (lip10) and ultimately to class II-associated Ii peptide (CLIP), which remains in the peptide-binding groove [47, 48]. Then, CLIP is removed from the class II molecules catalyzed by the human leukocyte antigen DM which is capable of binding with antigenic peptides. Subsequently, the CLIP complex is transported to the cell surface, on which the antigen is presented to cognate T cells, initiating an immune response [49]. The naïve CD4^+^ T helper (Th0) cells can differentiate into CD4^+^ cell subtypes, including Th1, Th2, Th17, and regulatory T cells (Tregs), depending on the cytokines which are produced. In the presence of IL-12, Th1 cells are derived by IFN-*γ* and TNF-*α*, while Th2 cells are derived in the presence of IL-4, which produce IL-4, IL-5, and IL-10. Th17 cells, which secrete IL-17, IL-23, and IL-22, are derived in the presence of TGF-*β*, IL-1*β*, and IL-6. In contrast, Tregs are raised in the presence of TGF-*β*, which secretes the immunosuppressive cytokines IL-10 and TGF-*β*. In the participation of immune responses, IL-17 stimulates the production of inflammatory mediators, including TNF-*α*, IL-6, and IL-1*β*, while Tregs effectively regulate the resolution of inflammation [50, 51]. An in vitro study has reported that CD4^+^ T cells with its proinflammatory cytokines IFN-*γ* and IL-6 serve as important routes for alveolar bone resorption in mice infected with *P. gingivalis* [46]. Tregs and Th17 cells have been demonstrated in periodontal tissues which are involved in periodontal disease processes [52]. Th17 cells promote bone destruction [51, 53], while Tregs protect alveolar bone by inhibiting osteoclastogenesis [54]. Therefore, the plasticity and cross-talk in T-cell subsets are vital for the regulation of the cellular immune response during periodontitis and therapeutic strategies comprising Tregs inhibiting the immuno-inflammatory response and restoring alveolar bone homeostasis [55]. Indeed, the effect of antibiotic therapy in regulating Treg-Th17 plasticity in humans with periodontitis is demonstrated [56].

## 3. Involvement of Cathepsin B in Innate Immune Responses

### 3.1. CatB in Periodontitis

CatB, functioning as an endopeptidase at neutral pH, is also found in the extralysosomal sites involving the cytosol, plasma membrane, and pericellular spaces [57]. In the noninvasive diagnostic body fluid, gingival crevicular fluid (GCF), CatB was detected mainly in macrophages when monocytes were migrating into the gingival crevice [58, 59]. The protease activity of CatB in GCF is closely associated with the GCF volume and the severity of periodontitis [60, 61]. CatB in GCF plays a major role in the pathology of periodontitis with respect to connective tissue breakdown and bone resorption. It is demonstrated that there is an imbalance between cathepsin B and the endogenous inhibitor cystatin C, with the elevated level of cathepsin B and a decreased level of cystatin C [62]. As a result of the proteinases from *P. gingivalis* infection, the hemostasis of CatB activity is disrupted, contributing to the destruction in periodontitis [63]. Therefore, the activity and the amount of cathepsin B and cystatin C in GCF can potentially serve as a predictor of attachment loss and an indicator of the progression of the disease [61, 64, 65]. CatB plays a significant role in promoting chronic inflammation in periodontitis. It is reported that CatB regulates the expression of collagens III and IV by fibroblasts in response to a TLR2 agonist, *P. gingivalis* LPS [66]. Moreover, CatB recently has been determined to be involved in the production of amyloid *β*, the main component of the hall marker in the brain of AD patients, in the macrophages in the gingival tissues of the patients with periodontitis and in the liver of mice after chronic systemic *P. gingivalis* infection [67].

### 3.2. CatB in IL-1*β* Processing

IL-1*β* is recognized as the master mediator in innate immune responses, and two signals are required for IL-1*β* processing. One is NF-*κ*B-dependent pro-IL-1*β* production, and the other is caspase-1-dependent proteolytic processing of pro-IL-1*β* to mature IL-1*β*. CatB plays a critical role in both signals of IL-1*β* processing because CatB involves in NF-*κ*B activation [68] and activation of caspase-1 [6, 7]. We have demonstrated that CatB was colocalized with caspase-1, and treatment with CA-074Me, a specific CatB inhibitor, markedly inhibited caspase-1 expression, resulting in a decreased production of IL-1*β* [69–71]. These findings are consistent with the observations that IL-1*β* and CatB are colocalized in phagolysosomes, and that the secretion of IL-1*β* is through the exocytosis of phagolysosomes in LPS-activated human monocytes [72]. The CatB expression is upregulated in the macrophages which involves the production of IL-1*β* and amyloid *β* in gingival tissues of the patients with periodontitis [67, 73]. Furthermore, CatB indirectly involves in the activation of caspase-1 through the proteolytic maturation of caspase-11 [74].

### 3.3. CatB in TNF-*α* Trafficking

Membrane-associated (m) TNF-*α* is a type II transmembrane precursor which is delivered from the *trans*-Golgi network to the recycling endosome [75, 76]. Membrane-associated TNF-*α* is transported to the cell surface, where it is cleaved by the TNF-*α*-converting enzyme [77]. Membrane fusion among the TNF-*α*-containing vesicles from the trans-Golgi network, the recycling endosomes, and the cell surface membrane is mediated by the interactions among various *trans*-soluble-*N*-ethylmaleimide-sensitive factor-attachment protein SNAP receptor (SNARE) family members [78]. Recent studies have revealed that the accumulation of newly synthesized mTNF-*α* originally occurs in the Golgi complex [79]. The mTNF-containing vesicles are then translocated from the *trans*-Golgi network to the recycling endosome and subsequently to the cell surface through two different membrane fusion processes [75, 76]. The first fusion process is mediated by Q-SNAREs, including syntaxin 6, syntaxin 7, and vesicle transport, through interacting with t-SNARE homolog 1b (Vit1b) of the Golgi complex TNF-*α* carrier vesicle and the R-SNARE vesicle-associated membrane protein 3 (VAMP3) of the recycling endosome. The second fusion process is mediated by the interactions between VAMP3 of the recycling endosome and the Q-SNARE complex which consists of syntaxin 4 and SNAP-23. LPS triggers expression of the Q-SNARE components, including syntaxin 4 and SNAP-23 for the accommodation of increased trafficking during TNF-*α* secretion in macrophages [80].

A recent report shows that cytosolic CatB is imperative for fusion of the TNF-*α*-containing vesicles to the plasma membrane [8]. CatB can be localized in the nucleus and downregulate transcriptions [81]. However, there is no difference in the levels of syntaxin 4 and SNAP-23 in the CA-074Me-treated or CatB−/− macrophages, suggesting that CatB does not downregulate the SNARE components. There is a possibility that CatB has functions in regulating TNF-*α* vesicle trafficking through regulation of the SNARE components, either at the transcriptional or posttranslational levels in THP-1 and primary human monocytic cells considering that CatB was reported to be involved in both transcription and posttranslational protein processes, such as protein [82] and thyroglobulin [83]. A new role for intracellular CatB activity involved in TNF-*α* signaling is suggested in the report [8].

Therefore, CatB plays a critical role in regulating innate immune responses in periodontitis by controlling production of IL-1*β* and TNF-*α*.

## 4. Involvement of Cathepsin S in Adaptive Immune Responses

### 4.1. CatS in Periodontitis

The general role of CatS is breaking down antigenic and antimicrobial peptides, involving antigen processing and presentation [84–86]. In addition, CatS also serves as an elastase, destroying extracellular matrix proteins, comprising collagen and proteins of the bacterial outer membrane [84, 87]. CatS is generated by immune cells, including macrophages and DCs [88, 89]. In an analysis of healthy buccal gingival tissues from Rhesus monkeys, the transcription of CatS was altered in aged healthy tissues compared with younger animals. These age-related immune pathways were associated with periodontal health [90]. CatS was also found to be upregulated in rats with experimental periodontitis and human patients with periodontitis [11, 91]. CatS is recognized as one of the hub proteins in the protein-protein interaction network of 726 differentially expressed genes in periodontitis and plays an essential role in bone loss involved in periodontitis progression [58, 91].

### 4.2. CatS in MHC II Antigen Presenting and CD4^+^ T-Cell Activation

CatS is essential in MHC II antigen processing and presentation [92, 93]. Indeed, CatS null mice show a considerable variation in generation of MHC II-bound Ii fragments, and presentation due to the fact that the Ii degradation in professional APCs is substantially diminished, where CatS is abundantly expressed [94, 95]. In addition, endocytosis selectively targets exogenous material to CatS in human DCs [96]. Enrichment of MHC II molecules within late endocytic structures has consistently been seen in splenic DCs of CatS-deficient mice [97]. Pharmacological or genetic inhibition of CatS results in defective TLR2 signaling in the *P. gingivalis* LPS-exposed DCs, indicating CatS may be involved in innate immune responses [10, 42].

### 4.3. CatS in Promoting of Th1 and Th17

Cathepsin S, which is predominantly generated by monocytes/macrophages and DCs, is involved in the ultimate proteolytic cleavage stage of the invariant chain in APCs during TLR2 signaling [48, 85, 88]. DCs induce the differentiation of naïve CD4^+^ T cells into various subpopulations, comprising Th1 (IFN-*γ*) or Th17 (Th17) [98, 99]. Recently, we reported that CatS is crucial for the differentiation of naïve CD4^+^ T cells [10]. We have found that CatS deficiency significantly relieves lower Th1 cell responses, accompanied with a decreased level of IFN-*γ* [10]. Moreover, we have demonstrated that CatS promotes differentiation of Th17 cells in response to *P. gingivalis* LPS-exposed DCs through protease-activated receptor (PAR) 2-dependent IL-6 production [42]. Subsequently, the increased IL-6 triggers the differentiation of Th17 cells, illustrating the significant role of CatS in the differentiation of Th17 cells, after the exposure to *P. gingivalis* LPS [42]. Considering the critical roles of CatS in adaptive immune responses through promoting the differentiation of both Th1 and Th17 cells in periodontitis, CatS may provide a novel therapeutic target for treatment of periodontitis.

## 5. Involvement of Cathepsin K in Immune Responses

### 5.1. CatK in Periodontitis

Cathepsin K (CatK) is dominantly generated by osteoclasts, which serves as an important regulator for bone resorption [100]. CatK is essential for normal bone resorption via degrading collagens and gelatin, the latter being a hydrolysis product of collagen [101, 102]. And it also dissolves type I collagen, the major component of the organic bone matrix [103]. Recently, several studies have demonstrated that CatK plays a crucial role in alveolar bone resorption in patients with periodontitis. It is reported that there are elevated levels of CatK in GCF of patients with periodontitis and periimplantitis [104–107]. Once the hemostasis is destroyed, bone resorption will occur in periodontitis. In patients with chronic periodontitis, CatK levels were greatly elevated in smokers compared to nonsmokers, indicating a positive influence of smoking on CatK [108]. Moreover, CatK in GCF is not only derived from osteoclasts, but also from fibroblasts, macrophages, and gingival epithelial cells, contributing to the attachment loss and alveolar bone resorption [109]. Pharmacologic or genetic inhibition of CatK results in defective TLR9 signaling in DCs in response to unmethylated CpGDNA, without affecting antigen-presenting ability [110].

### 5.2. CatK in Autophagy

The elevated level of CatK not only directly induces bone resorption, but also makes impact on innate immune indirectly contributing to periodontitis and even autoimmune diseases, especially rheumatoid arthritis (RA) [9]. As the level of CatK is elevated, there would be an activated TLR9 signaling in DCs and macrophages and consequently, osteoclastic bone resorption [103, 110]. The CpGDNA can be recognized by TLR9, which can trigger autophagy [111–113]. In response to the activation of TLR9, the autophagy protein microtubule-associated protein 1A/1B‐light chain 3 (LC3) aggregates I*κ*B kinase *α* (IKK*α*) for type I interferon generation [112]. Moreover, there is a significant increase in the TLR9 downstream proteins (IKK*β* and MYD88) and TFEB, which are greatly correlated with autophagy, in response to the activation of TLR9 [114, 115]. Coinciding with the conclusion that the activation of TLR9 can induce the downstream autophagy pathway, the inhibition of CatK can suppress this response via downregulating the expression of TLR9 [9, 116]. Therefore, CatK may involve in innate immune responses in periodontitis through the TLR/autophagy pathway.

## 6. Conclusion

**Figure 1:**
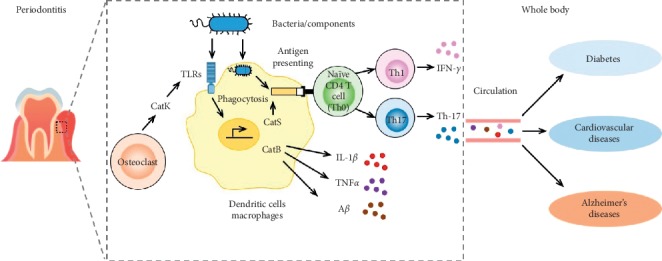
A schematic illustration of the cathepsin-related immune responses in periodontitis linking to the diseases within the whole body. In the bacteria (components)-stimulated cells, cathepsin B involves in activation of TLR signaling to produce IL-1*β*, TNF-*α*, and amyloid (A) *β*, and cathepsin S involves in maturation of MHC class II for driving CD4^+^ (helper) T cells to produce IFN-*γ* (Th1) and IL-17 (Th17). Cathepsin K involves in activation of the TLR/autophagy pathway to produce type I interferon (IFN). The periodontitis cathepsin-related proinflammatory mediators involve in systemic diseases and Alzheimer's disease via the circulation.

The involvement of cathepsins in the immune responses of periodontitis contribute to systemic diseases, including diabetes, cardiovascular diseases, and neurodegenerative disease, including AD, especially increasing in aged population (Figure 1). We believe that regulation of cathepsins, including CatB, CatS, and CatK, in cellular immune responses in patients with periodontitis will be beneficial for reduction of systemic diseases and neurodegenerative diseases in the global aging society.
